# Association of Diabetes Mellitus with a Combination of Vitamin D Deficiency and Arsenic Exposure in the Korean General Population: Analysis of 2008–2009 Korean National Health and Nutrition Examination Survey Data

**DOI:** 10.1186/2052-4374-25-7

**Published:** 2013-05-21

**Authors:** Byung-Kook Lee, Yangho Kim

**Affiliations:** 1 Institute of Environmental & Occupational Medicine, Soonchunhyang University 646 Eupnae-ri, Shinchang-myun, Asan-si, Choongnam, 336-745, South Korea; 2 Department of Occupational and Environmental Medicine, Ulsan University Hospital, University of Ulsan College of Medicine, Ulsan, South Korea

**Keywords:** Arsenic, Diabetes, Vitamin D, 25-hydroxyvitamin D

## Abstract

**Objectives:**

We present data from the Korean National Health and Nutritional Examination Survey (KNHANES) 2008–2009 on the combination of vitamin D deficiency and arsenic exposure on diabetes mellitus (DM) in a representative sample of the adult Korean population.

**Methods:**

This study was based on data obtained from the KNHANES 2008–2009, which was conducted for 3 years (2007–2009) using a rolling sampling design that involved a complex, stratified, multistage, probability-cluster survey of a representative sample of the non-institutionalized civilian population in South Korea.

**Results:**

Data analysis revealed that subjects who showed both vitamin D levels in the 1^st^ quartile (Q) and urinary arsenic levels in the 4^th^ Q, had a 302% increased risk of having DM, as compared with those whose vitamin D and urinary arsenic levels were in the 4^th^ Q and 1^st^ Q, respectively.

**Conclusion:**

The present study reconfirmed an association of DM with low vitamin D levels and arsenic exposure, and further showed a combination of vitamin D deficiency and arsenic exposure on DM in the general Korean population. To the best of our knowledge, this is the first report describing a combination of vitamin D deficiency and arsenic exposure on DM. The present findings have important public health implications.

## Introduction

Socioeconomic development and changes in nutrition in the Republic of Korea between the late 1980s and 2005 were accompanied by an increase in the prevalence of type 2 diabetes, from 3% to 7.3%, and increased concern about diabetes mellitus (DM) [1]. Increasing rates of DM worldwide suggest that the condition is related to environmental factors [2].

In epidemiological studies from Taiwan, Bangladesh, and Mexico, high chronic exposure to inorganic arsenic in drinking water (100 μg/L) has been shown to be associated with DM [3]–[9]. High chronic exposure to inorganic arsenic in occupational settings was also related to higher levels of glycated hemoglobin, a marker of blood glucose levels [10]. The effect of lower levels of exposure to inorganic arsenic on DM risk has also been reported [2,4,11,12]. In addition, our recent study showed an association between urinary arsenic and DM in the general Korean population [13].

Vitamin D plays an important role in bone and mineral metabolism, and its deficiency is closely associated with the occurrence of metabolic bone disease, such as rickets in children and osteomalacia in adults. Recently, vitamin D has been drawing the interest of medical researchers due to its potential role in DM [14,15]. Several studies have suggested a link between low vitamin D status as measured by 25-hydroxyvitamin D [25(OH)D] and the occurrence of DM [16]–[20].

Both arsenic exposure and vitamin D deficiency are commonly encountered public health issues [11,21]–[25]. However, the combined effect of vitamin D deficiency and arsenic exposure on DM has never been reported. We hypothesized an association of DM with a combination of vitamin D deficiency and arsenic exposure in the general Korean population. To test this hypothesis, we conducted a cross-sectional analysis on the association of vitamin D and arsenic levels with DM status using the Korean National Health and Nutritional Examination Survey (KNHANES) 2008–2009 data set. Our study objective was to evaluate whether there is an association of DM with the combination of vitamin D deficiency and arsenic exposure in a representative sample of the adult Korean population.

## Materials and Methods

### Design and data collection

This study was based on data obtained from the KNHANES 2008–2009, which represents the second and third years of the KNHANES IV 2007–2009 survey. This survey was conducted for 3 years (2007–2009) using a rolling sampling design that involved a complex, stratified, multistage, probability-cluster survey of a representative sample of the non-institutionalized civilian population in South Korea. Detailed information on the design was provided previously [26]. Briefly, the survey consisted of three components: a health interview survey, a health examination survey, and a nutrition survey. For the present analysis, we used data from a total of 3393 adults (age ≥ 20 years) who participated in the health examination survey, had documented urinary arsenic measurements, and completed the nutrition survey. All participants provided written consent to participate in the study.

The Institutional Review Board requirement was waived because the KNHANES was conducted by the Korean government in accordance with the internationally agreed-upon ethical principles for the conduct of medical research.

Information on age, education, smoking history, alcohol intake, and regular exercise or walking was collected during the health interview. Height and weight measurements were performed with the participants wearing light clothing and no shoes. The body mass index (BMI) was calculated as weight in kilograms divided by the square of the height in meters. The BMI was categorized into three groups: lean (BMI < 18.5), normal (18.5 ≤ BMI < 25), and obese (BMI ≥ 25). Age as reported at the time of the health interview was categorized into six groups. Residence area was categorized into urban areas (administrative divisions of a city) and rural areas (not classified as administrative divisions of a city).

Education level was categorized into three groups: below high school, high school, and some college or higher. Smoking status was divided into three categories: current smoker, past smoker, and never-smoker. Smoking status was defined based on self-reported cigarette use: Never-smokers had smoked fewer than 100 cigarettes in their lifetime and participants who had smoked 100 or more cigarettes were classified as past or current smokers based on current use. Alcohol consumption was assessed by questioning the participants about their drinking behavior during the month prior to the interview. The participants were asked about their average frequency (days per month) of alcoholic beverage consumption and amount (in mL) of alcoholic beverages ingested on a single occasion. The responses were converted into the amount of pure alcohol (in g) consumed per day. The alcohol consumption status was categorized into four groups according to average daily alcohol consumption: non-drinker, light drinker (1–15 g), moderate drinker (16–30 g), and heavy drinker (> 30 g).

Information regarding the frequency of seafood consumption, including fish, shellfish, and seaweed, was obtained from the KNHANES 2008 nutrition survey, which was performed separately on different dates after the health examination. The nutrition survey listed 11 types of seafood that are consumed most frequently in Korea: mackerel, tuna, yellow fish, pollock, anchovies, seafood paste, squid, clams, pickled seafood, brown seaweed, and laver. The overall consumption frequency was categorized into three groups based on the consumption of at least one type of seafood on the nutrition survey checklist: less than once a week, once a week, and more than once a week.

Regular walking was defined as walking for 30 or more minutes at a time at least five times per week, regardless of indoors or outdoors. Regular exercise was defined as moderate exercise (swimming slowly, playing doubles tennis, volleyball, occupational or recreational activities of carrying light objects) on a regular basis for 30 or more minutes at a time at least five times per week or vigorous exercise (running, climbing, cycling fast, swimming fast, football, basketball, jump rope, squash, playing singles tennis, and occupational or recreational activities of carrying heavy objects) for 20 or more minutes at a time at least three times per week. The seasons were classified into four categories as follows: spring (March to May), summer (June to August), fall (September to November), and winter (December to February). DM was defined as a fasting glucose level ≥ 126 mg/dl, current use of anti-diabetic medications, or self-reported physician diagnosis of DM.

### Measurement of serum 25-hydroxyvitamin D [25(OH)D)]

For the measurement of serum 25(OH)D, blood samples of individual participants were collected during the health examination survey. The blood samples were centrifuged, aliquoted, and frozen to −70°C on site. The frozen serum samples were transported on dry ice to the designated central laboratory of Neodin Medical Institute, a laboratory certified by the Korean Ministry of Health and Welfare in Seoul, Korea.

Blood samples were analyzed within 24 hours after transportation. Serum 25(OH)D levels were measured using a gamma counter (1470 Wizard, Perkin-Elmer, Finland) with a radioimmunoassay (RIA) kit (DiaSorin, Stillwater, MN). Fasting blood samples were taken in the morning after at least an 8-h fast. Plasma glucose levels were measured using an autoanalyzer (Hitachi 7600 autoanalyzer; Hitachi, Tokyo, Japan).

### Measurement of urinary arsenic (μg/g creatinine)

Spot urine samples for total arsenic in urine were collected at the time of health checkups. Total arsenic was measured in 0.1-mL samples of urine by graphite furnace atomic absorption spectrometry with Zeeman background correction (Perkin Elmer AAS 600; Perkin Elmer, Singapore) [27]. All analyses of total urinary arsenic were creatinine-adjusted and performed by Neodin Medical Institute. For internal quality assurance and control, commercial reference materials were obtained from Bio-Rad (Lyphochek® Whole Blood Metals Control; Bio-Rad, Hercules, CA). The coefficients of variation were within 0.57%–3.20% for three reference samples of total arsenic (reference values: 66.0, 160.0, and 278.0 μg/L). As part of external quality assurance and control, the institute passed the German External Quality Assessment Scheme operated by Friedrich-Alexander University and also passed the Quality Assurance Program operated by the Korea Occupational Safety and Health Agency. The institute also held a certified license from the Ministry of Labor as one of the designated laboratories for special chemicals, including heavy metals and certain organic chemicals. The method detection limit for total arsenic in urine in the present study was 2.584 μg/L. None of the urine samples had arsenic levels below the limit of detection.

### Statistical analysis

Statistical analyses were performed using SAS (Version 9.22, SAS Institute, Cary, NC, USA) and SUDAAN (Release 10.0, Research Triangle Institute, Research Triangle Park, NC, USA), a software package that incorporates sample weights and adjusts analyses for the complex sample design of the survey. Survey sample weights were used in all analyses to produce estimates that were representative of the non-institutionalized civilian Korean population.

The adjusted means [95% confidence interval (CI)] of serum 25(OH)D and urinary arsenic were calculated by sex, age group, residence location, season, educational level, smoking status, drinking status, BMI, regular exercise, regular walking, seafood consumption, and DM after adjustment for all other variables in the analysis using the Proc Regress function in SUDAAN.

Next, the odds ratio (OR) and 95% CI values for having DM were calculated for three models using categories containing a combination of quartiles of 25(OH)D and urinary arsenic concentration (AsU) while controlling for age, BMI, sex, residence location, season, education level, smoking and drinking status, regular exercise and walking, and seafood consumption. The employed models were as follows:

Model 1: quartiles of 25(OH)D

Model 2: quartiles of AsU

Model 3: combination of quartiles of 25(OH)D and AsU: group 1): 25(OH)D 4th Q and AsU 1st Q; group 2): 25(OH)D 1st Q and AsU 4th Q.

**Figure 1:**
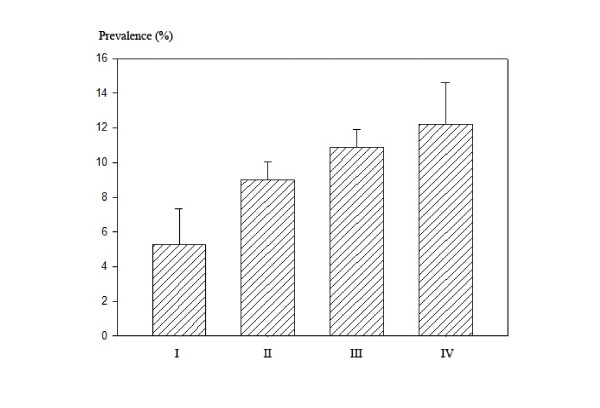
**Prevalence (%) of diabetes mellitus in four groups classified by quartiles of vitamin D and urinary arsenic (AsU).** (I: 4th Q of vitamin D and 1st Q of AsU, II: 1st Q of vitamin D, III: 4th Q of AsU, IV: 1st Q of vitamin D and 4th Q of AsU).

The adjusted prevalence (%) and 95% CI were also obtained using the predicted marginal calculation after the same covariate adjustments for logistic regression analysis from Proc RLOGIST of SUDAAN and are presented in Figure 1, which depicts the graphical comparison of four different groups categorized by a combination of quartiles of 25(OH)D and AsU.

## Results

The arithmetic mean values and 95% CI of serum 25(OH)D and the geometric mean values and 95% CI of AsU of the study participants are listed in Table 1 by the categories of sex, age, residence location, season, education level, smoking and drinking status, BMI, regular exercise and walking, seafood consumption, and DM. The mean values were adjusted for all other variables in the table. Overall, the mean serum 25(OH)D and AsU for women (*n* = 1780) were 42.8 nmol/L (95% CI, 41.6–44.2 nmol/L) and 128.7 μg/g creatinine (95% CI, 122.8–134.8 μg/g creatinine), respectively, and the mean levels for men (*n* = 1613) were 49.8 nmol/L (95% CI, 48.3–51.3 nmol/L) and 101.1 μg/g creatinine (95 CI, 96.4–106.1 μg/g creatinine), respectively. The serum 25(OH)D level was significantly higher in men than in women, but the AsU was significantly lower in men than in women. Both levels were significantly higher with increasing age. The mean 25(OH)D levels of individuals living in rural areas were significantly higher than those in urban areas, but no difference was observed in the AsU between urban and rural areas. Individuals tested in the spring and winter, the seasons with the lowest amount of sun exposure, had significantly lower serum 25(OH)D levels than individuals tested in the seasons with the greatest amount of sun (i.e., summer and fall). On the other hand, the mean AsU levels were significantly higher in the winter as compared to the spring. While education level and BMI did not reveal any mean differences in either serum 25(OH)D or urinary arsenic, current smokers had significantly lower mean levels in both as compared with never-smokers. AsU levels were significantly higher in people who drink heavily as compared to those who did not drink. The mean levels of 25(OH)D were higher in people who walked or exercised regularly, and the mean levels of urinary arsenic were higher in people who ate more seafood. No mean difference in 25(OH)D was observed between diabetic statuses, while a significantly higher mean level of AsU was observed in diabetic subjects.

**Table 1:** Serum 25(OH)D and Urinary arsenic concentration, adjusted for all other variables in the table

**Variables**	**N**	**Serum 25(OH)D**		**Urinary arsenic**	
		**AM (95% CI)**	**p-value**	**GM (95% CI)**	**p-value**
Sex					
Men	1613	49.84 (48.34-51.34)	*	101.1 (96.42-106.1)	*
Women	1780	42.88 (41.62-44.15)	0.000	128.7 (122.8-134.8)	0.000
Age (years)					
20-29	630	41.89 (40.12-43.66)	*	69.77 (64.74-75.20)	*
30-39	677	45.20 (43.50-46.90)	0.001	100.6 (94.77-106.9)	0.000
40-49	671	45.86 (44.37-47.36)	0.000	127.1 (119.5-135.2)	0.000
50-59	686	49.30 (47.50-51.10)	0.000	141.0 (132.4-150.1)	0.000
60+	729	48.57 (46.51-50.63)	0.000	155.1 (145.2-165.6)	0.000
Residence location					
Urban	2616	44.81 (43.67-45.94)	*	114.4 (110.7-118.2)	*
Rural	777	51.30 (48.92-53.69)	0.000	116.9 (106.9-127.7)	0.671
Season					
Spring	905	44.78 (43.23-46.33)	*	108.3 (101.5-115.5)	*
Summer	788	47.42 (45.57-49.27)	0.017	113.8 (107.5-120.6)	0.222
Fall	534	46.88 (44.99-48.76)	0.055	116.6 (109.0-124.7)	0.126
Winter	1166	45.98 (44.60-47.35)	0.232	120.2 (113.9-126.9)	0.027
Education level					
Less than high school	1149	45.95 (44.15-47.76)	*	117.8 (110.9-125.2)	*
High school	1023	47.15 (45.72-48.58)	0.264	115.1 (109.1-121.4)	0.568
College and more	1221	45.48 (43.95-47.01)	0.706	112.4 (106.7-118.4)	0.289
Smoking status					
Never-smoker	1872	46.73 (45.45-48.02)	*	120.0 (114.9-125.3)	*
Past smoker	691	47.13 (45.22-49.03)	0.727	115.5 (108.1-123.4)	0.372
Current smoker	825	44.06 (42.28-45.83)	0.014	103.7 (97.56-110.3)	0.000
Drinking status					
No drink	905	44.78 (43.23-46.33)	*	108.3 (101.5-115.5)	*
Mild drink	788	47.42 (45.57-49.27)	0.017	113.8 (107.5-120.6)	0.222
Moderate drink	534	46.88 (44.99-48.76)	0.055	116.6 (109.0-124.7)	0.126
Heavy drink	1166	45.98 (44.60-47.35)	0.232	120.2 (113.9-126.9)	0.027
Body Mass Index					
Lean	153	45.89 (42.93-48.84)	*	108.7 (96.52-122.4)	*
Normal	2162	46.12 (44.98-47.26)	0.876	114.8 (110.7-119.1)	0.382
Obese	1047	46.23 (44.92-47.54)	0.827	116.1 (110.5-122.1)	0.325
Regular exercise					
Yes	534	48.95 (47.07-50.84)	*	111.4 (104.0-119.4)	*
No	2859	45.62 (44.59-46.65)	0.000	115.6 (111.8-119.5)	0.351
Regular walking					
Yes	1629	47.14 (45.89-48.40)	*	115.0 (110.6-119.5)	*
No	1764	45.24 (44.06-46.42)	0.006	114.8 (110.3-119.5)	0.952
Seafood consumption					
Less than once a week	310	47.76 (45.42-50.09)	*	103.5 (93.93-114.0)	*
Once a week	1031	45.47 (44.11-46.83)	0.069	111.0 (105.5-116.9)	0.223
More than once a week	2052	46.26 (45.11-47.41)	0.224	118.7 (114.6-122.9)	0.007
Diabetes					
Yes	309	44.46 (42.00-46.91)	*	128.9 (117.3-141.8)	*
No	3084	46.30 (45.26-47.35)	0.148	113.7 (110.2-117.2)	0.011

*: reference group; AM, arithmetic mean; GM, geometric mean.

Subjects whose 25(OH)D levels were in the 1st Q category had a tendency toward an increased risk of having DM as compared with those whose 25(OH)D levels were in 4th Q although the difference was not statistically significant (model 1)(Table 2). The risk of having DM was 1.65 times higher in the group categorized as having urinary arsenic in the 4th Q, as compared with those who were in the category of AsU in the 1st Q (model 2). The risk of having DM was 3.02 times higher in the group categorized as 25(OH)D in the 1st Q and urinary arsenic in the 4th Q, as compared with those who were in the category of 25(OH)D in the 4th Q and AsU in the 1st Q (model 3).

**Table 2:** Adjusted OR (95% CI) of Korean adults having diabetes mellitus by category of combination of 25-hydroxyvitamin D quartile and urinary arsenic quartile, adjusted for covariates#

**Model**	**Independent variables**		**Odds ratio**	**95% Confidence Interval**
Model 1				
	Age (years)		1.060	1.046~1.074
	Body mass index (kg/m^2^)		1.162	1.108~1.218
	Sex			
	Men		Ref	
	Women		1.053	0.697~1.351
	Quartile of serum 25-hydroxyvitamin D			
	1. 1st Q ( ≤ 30.02)		1.250	0.827~1.888
	2. 2nd Q (31.02 < Q ≤ 44.11)		1.395	0.931~2.091
	3. 3rd Q (44.11 < Q ≤ 55.99)		1.216	0.804~1.838
	4. 4th Q ( > 55.99)		Ref	
Model 2				
	Age (years)		1.057	1.042~1.071
	Body mass index (kg/m^2^)		1.163	1.108~1.220
	Sex			
	Men		Ref	
	Women		1.015	0.679~1.518
	Quartile of urinary arsenic			
	1. 1st Q ( ≤ 69.67)		Ref	
	2. 2nd Q (69.67 < Q ≤ 114.86)		1.056	0.646~1.727
	3. 3rd Q (114.86 < Q ≤ 159.48)		1.200	0.742~1.940
	4. 4th Q ( > 159.48)		1.654	1.038~1.518
Model 3				
	Age (years)		1.074	1.026~1.123
	Body mass index (kg/m^2^)		1.260	1.134~1.400
	Sex			
	Men		Ref	
	Women		0.776	0.239~2.519
	Combination of Vitamin D & AsU			
	1. Vitamin D 4thQ + AsU 1stQ		Ref	
	2. Vitamin D 1stQ + AsU 4thQ		3.023	1.004~9.105

#Covariates: residence location (urban or rural), season, education level, smoking & drinking status, regular exercise & walking and seafood consumption; Q, quartile; AsU, urinary arsenic (g/g creatinine).

The adjusted prevalence (%) and 95% CI of DM were also obtained using predicted marginal calculations, and are presented in Figure 1 to depict the graphical comparison of the four different groups categorized with a combination of quartiles of 25(OH)D and AsU levels. Those whose 25(OH)D levels were in the 4th Q and AsU were in the 1st Q had a 5.29% prevalence of DM, whereas those in the worst group with levels in the 1st Q of 25(OH)D and 4th Q of AsU had a 12.19% prevalence of DM, respectively.

## Discussion

The present study focused on vitamin D deficiency and arsenic exposure, which are environmental risk factors for DM [4,11]–[20] and are commonly encountered public health issues [8,11,21]–[23,25].

In the present study, subjects whose 25(OH)D levels were in the 1st Q and whose AsU levels were in the 4th Q had a 302% increased risk of having DM as compared with those whose 25(OH)D and AsU levels were in the 4th Q and 1st Q, respectively. The overall prevalence of DM in our study population was 9.11% (309/3393). From predicted marginal calculations, the adjusted prevalence ranged from 5.29 to 12.19%, depending on the levels of 25(OH)D and AsU. The present study reconfirmed an association of DM with low vitamin D levels and arsenic exposure, and further showed an association of DM with the combination of vitamin D deficiency and arsenic exposure in the general Korean population. To the best of our knowledge, this is the first report describing an association of DM with the combination of vitamin D deficiency and arsenic exposure.

Animal and in vitro model systems have indicated that arsenic exposure can potentially increase the risk of DM through inhibition of insulin-dependent glucose uptake [28] and insulin signaling [29], impairment of insulin secretion and transcription in pancreatic beta cells [30], and modification of the expression of genes involved in insulin resistance [30]. The mechanisms underlying the potential association between DM and 25(OH)D are unclear. The 25(OH)D level may have an impact on many factors, including pancreatic beta cell function, insulin action, and systemic inflammation [31]. Accumulating evidence indicates that vitamin D may be useful in the prevention and treatment of DM. Vitamin D supplementation in participants with vitamin D deficiency has been shown to result in improved glucose tolerance [32]. The mechanisms underlying the potential association of DM with the combination of 25(OH)D and arsenic remain to be clarified.

The present findings have several implications with respect to environmental health. First, the present results indicate the importance of assessing vitamin D levels or arsenic exposure when addressing their association with DM. Second, vitamin D deficiency may be critical for assessing the risk of DM in people living in regions in which arsenic exposure is endemic, or in the vicinity of highly contaminated areas, such as abandoned metal mines without strict environmental controls, or arsenic-emitting factories. Considering the high prevalence of vitamin D deficiency [21]–[23], these findings have important public health implications in the general population.

The present study had several important strengths. First, we used serum 25(OH)D concentration as an indicator of vitamin D levels, which reflects vitamin D obtained from diet, supplements, and cutaneous synthesis. Second, relevant DM risk factors and confounders, including seafood consumption, were adjusted for. Third, the study was performed in a representative sample of the general Korean population. Finally, rigorous quality control of the study procedures was ensured in the KNHANES.

The present study had some limitations. First, our associations were obtained from a cross-sectional analysis. We cannot completely exclude the possibility of reverse causality. A third factor might be a common link which produces the association: Low physical activity might be associated with both DM and low vitamin D. Therefore, a causal relationship between serum vitamin D level and arsenic level and DM cannot be inferred. A prospective study, in which arsenic or vitamin D levels are determined before the development of disease, will be required to establish the causal nature of these associations.

Second, data on sun exposure and dietary vitamin D intake were not available. Instead, we obtained data regarding the participants’ exercise and walking habits, which presumably provide an estimate of each subject’s sunlight exposure [33]. We also adjusted for assumed seasonal exposure. Third, we did not perform species analysis of arsenic and could not rule out contributions of organic arsenic such as arsenobetaine and arsenosugars mainly derived from seafood to total AsU levels [13,34,35]. Instead, we adjusted for seafood consumption as the main sources of organic arsenic. Fourth, family history is a very important risk factor for DM; however, these data were not available in the 2008–2009 KNHANES.

## Conclusion

In conclusion, we showed an association of DM with the combination of vitamin D deficiency and arsenic exposure in the general Korean population.

## Competing interests

The authors declare that they have no competing interests.

## Authors’ contributions

All authors had access to the data and played a role in writing this manuscript: study concept and design (YK); acquisition, statistical analysis and interpretation of data (B-KL); drafting of the manuscript (YK); critical revision of the manuscript (B-KL); All authors read and approved the final manuscript.
